# Remapping and Reconnecting the Language Network after Stroke

**DOI:** 10.3390/brainsci14050419

**Published:** 2024-04-24

**Authors:** Victoria Tilton-Bolowsky, Melissa D. Stockbridge, Argye E. Hillis

**Affiliations:** Departments of Neurology, Physical Medicine & Rehabilitation, and Cognitive Science, Johns Hopkins University School of Medicine, Baltimore, MD 21287, USA; vbolows1@jhmi.edu (V.T.-B.); md.stockbridge@jhmi.edu (M.D.S.)

**Keywords:** stroke, aphasia, mechanisms of recovery, language networks, connectivity

## Abstract

Here, we review the literature on neurotypical individuals and individuals with post-stroke aphasia showing that right-hemisphere regions homologous to language network and other regions, like the right cerebellum, are activated in language tasks and support language even in healthy people. We propose that language recovery in post-stroke aphasia occurs largely by potentiating the right hemisphere network homologous to the language network and other networks that previously supported language to a lesser degree and by modulating connection strength between nodes of the right-hemisphere language network and undamaged nodes of the left-hemisphere language network. Based on this premise (supported by evidence we review), we propose that interventions should be aimed at potentiating the right-hemisphere language network through Hebbian learning or by augmenting connections between network nodes through neuroplasticity, such as non-invasive brain stimulation and perhaps modulation of neurotransmitters involved in neuroplasticity. We review aphasia treatment studies that have taken this approach. We conclude that further aphasia rehabilitation with this aim is justified.

## 1. Introduction

Aphasia refers to deficits in language (comprehension and production, written and spoken) following damage to the brain. It is distinct from deficits in broader cognition or articulation (dysarthria, apraxia). Recovery of language function after a stroke causing aphasia is thought to take place in part through “reorganization” of structure–function relationships or “take-over” (by undamaged) tissue of functions that are impaired by damaged tissue. One interpretation of this concept is that neurons are sufficiently pluripotent; that is, they can change the type of stimulus they are tuned to or that a functional network can change the type of computation it carries out. Makin and Krakauer [1] review extensive evidence from animal and human studies against this interpretation of reorganization. They argue instead that remapping occurs through potentiation (i.e., increases in synaptic efficacy or strengthening of synapses through activity) of preexisting networks or circuits that have the necessary representational and computational capacity prior to stroke. Potentiation of preexisting networks that may have been supportive of function such as language can be facilitated via Hebbian learning and other neuroplasticity mechanisms. Hebbian learning mechanisms are engaged through repeated patterns of neuronal firing, which is thought to strengthen these pathways and make them more efficient [2]. Neuroplasticity refers to the brain’s ability to form new connections and/or reorganize to restore or regain function after some disruption in function. While Makin and Krakauer mention language recovery, their paper focuses on motor and sensory recovery after injury.

In this paper, we similarly propose that language recovery takes place largely through remapping language networks by potentiating the right-hemisphere network homologous to the language network (hereafter referred to as the “right-hemisphere language network”) and by modulating connection strength between nodes of the right-hemisphere language network and undamaged nodes of the left-hemisphere language network. Based on this premise (supported by evidence we review), we propose that interventions should be aimed at potentiating the right-hemisphere language network through Hebbian learning or by augmenting connections between network nodes through neuroplasticity (such as non-invasive brain stimulation and perhaps modulation of neurotransmitters involved in neuroplasticity). The aim of our discussion focuses on evidence from the most common patterns of hemispheric functional dominance observed in the population. In the majority of healthy people, functional representations unique to higher-level language processing are predominantly left-lateralized, while lower-level processing underpinning language, such as sound identification, is more commonly associated with a bilateral representation [3].

First, we review a representative sample of evidence from neurotypical control participants for the existence of a reliable language network in the left hemisphere as well as a homologous right-hemisphere language network that together support virtually all language functions, including phonological (sound based), orthographic (writing based), semantic (meaning based), and syntactic (grammar based) processes involved in understanding and producing spoken and written language. Certainly, an exhaustive review is impossible in a single paper. However, we review illustrative studies using various functional resting state and task-related imaging approaches. These networks each include ventral (sound to meaning [3]) and dorsal (meaning to production [4]) streams [5] composed of cortical regions and their connections as well as the contralateral cerebellum [6]. Although these networks are modulated by subcortical structures such as the basal ganglia and thalamus, the role of subcortical structures in post-stroke aphasia is likely through diaschisis (i.e., dysfunction in distant areas of cortex that are otherwise spared but occurs due to their connections with damaged structures), and recovery from damage to these subcortical regions may reflect the resolution of diaschisis [7].

Then, we review evidence from functional imaging studies of people with aphasia indicating that recovery occurs through remapping and change in connection strength between nodes of the right- and left-hemisphere language networks as we have defined them. We include studies of positron emission tomography (PET), resting state and task-related functional magnetic resonance imaging (fMRI), and functional near-infrared spectroscopy (fNIRS), although there are also data from electroencephalography (EEG) that are relevant to the discussion.

Finally, we discuss the types of interventions that have been used in aphasia that might be utilized to potentiate networks that are supportive of language, including right-hemisphere language network and right cerebellar–cortical connections. We review interventions focused on enhancing the supportive roles of the right hemisphere in language processing through music, drawing, prosody, and manipulations to attention and intention. Although there is scant evidence that these interventions actually have potentiated, that is, increased, synaptic strength and efficiency via activity in the right-hemisphere language network, we provide directions for future studies to evaluate this hypothesis. We also review studies of treatments aimed at increasing neuroplasticity and connectivity between language network nodes, including connections between the right cerebellum and language network. Some of these studies have, in fact, demonstrated changes in connection strength as predicted by our proposals.

## 2. The Language Network and Supporting Areas in Neurotypical Controls

### 2.1. The Left-Hemisphere Language Network

One of the most remarkable findings from functional imaging of language processing is that the same cortical regions are activated in nearly every language task, even though damage to distinct regions causes very different deficits. Although “subtraction” designs, those selected to reveal distinct areas activated for two different language tasks (e.g., reading aloud irregular words minus reading aloud regular words), can show differences, virtually all language tasks activate the same regions of left hemisphere when contrasted with low level tasks that are primarily attentional or perceptual (e.g., fixation, counting, saying “skip” to scrambled pictures or scrambled words). Nodes of the language network generally include the posterior superior temporal cortex (pSTG, often referred to as “Wernike’s area”), middle temporal gyrus (MTG), inferior temporal gyrus or fusiform gyrus (FuG), posterior inferior frontal gyrus (pIFG, often referred to as “Broca’s area”), dorsolateral prefrontal cortex (DLPFC), and inferior parietal cortex (IPC), which includes the supramarginal gyrus (SMG) and angular gyrus (AG) (list of frequently-occurring abbreviations provided below). Importantly, recent authors have argued against viewing the language network as a set of discrete, specialized, regions each contributing a constituent function toward the emergence of language. Instead, it may be better understood as a synergistic network acting together [5,8,9].

Activation of this language network is observed in fMRI studies across clinical and healthy populations with tasks as divergent as word generation/letter fluency [10], word retrieval (naming and oral reading compared to counting) [11], comprehension and production of syntactically complex sentences [12], passive viewing and listening to discourse [13], detecting sensible vs. not sensible sentences [14], and reading [15]. Early PET studies first revealed activation in these areas during most language tasks, although PET studies also frequently showed activation of the cingulate cortex (see [16] and [17] for review). Occipital areas are activated consistently when visual stimuli are included as part of the task unless compared to a baseline condition that includes comparable visual demands.

This “language network” is among the networks revealed by task-free (“resting state”) fMRI. The best known (and first described) network of brain regions that show highly correlated blood oxygen level-dependent (BOLD) activation at rest is the Default Mode Network [18]. However, several other networks defined by their “connectivity” (correlated BOLD activity at rest) have been described, including the language network [19], which includes the network nodes described above, as well as superior frontal cortex.

Other types of studies have evaluated the interplay between nodes of the language network during specific tasks. For example, one study of concurrent transcranial magnetic stimulation and electroencephalography (TMS-EEG) revealed time- and region-specific causal evidence for a bidirectional flow of activation from the left pSTG/superior temporal sulcus (STS) to the left posterior inferior frontal gyrus (pIFG) and back during auditory sentence processing, as well as interplay between left pSTG/STS and left AG [20].

Structural imaging studies, for example, using diffusion tensor imaging (DTI), also have revealed the major white matter tracts that connect the nodes of the left-hemisphere language network [21,22]. In the dorsal stream of language processing (meaning to production), the three segments of the arcuate fasciculus with distinct connections and the frontal aslant tract provide the main connections within the language network. In the ventral stream, the connections are provided by the inferior longitudinal fasciculus, inferior fronto-occipital fasciculus, middle longitudinal fasciculus, uncinate fasciculus, and temporo-frontal extreme capsule fasciculus. The frontal aslant tract is a recently described short monosynaptic association tract connecting the lateral IFG to the superior frontal gyrus, an area that may have a supportive role in language, along with the cingulate cortex.

### 2.2. The Right-Hemisphere (Homologous) Language Cortex

The language network, as defined by task-free fMRI connectivity, also includes the right pSTG [19]. This finding fits well with current models of language processing that propose left-dominant dorsal and ventral streams of language processing, but also more bilateral processing of phonology in right and left pSTG. Virtually all fMRI studies of language processing by neurotypical controls show activation of at least some of the right hemisphere homologues of the language networks, although these areas are rarely discussed. For example, control participants presented with sensible sentences versus not sensible sentences activated right IFG, DLPFC, and MTG, as well as the left-hemisphere language network. Generally, activation of the right-hemisphere language network is lower than the left hemisphere homologues or may not include all of the language network [16,23]. While this may contribute to the trend of not acknowledging when bilateral activation is observed, it seems likely that a prepotent belief about hemispheric dominance also discourages investigators from interpreting right hemisphere activation as truly necessary to healthy language processing. When discussed, right hemisphere activation has sometimes been attributed to processing the prosody of language stimuli (e.g., emotional prosody [24]), recognizing multiple meanings of words (e.g., [25,26,27]), extracting the main idea or “gist” of discourse [28], or auditory processing of either the stimuli or one’s own spoken output.

Other studies have specifically evaluated the role of the right hemisphere in language tasks. For example, Patel and colleagues carried out an fMRI study of neurotypical participants producing and listening to discourse on a variety of topics [29]. They identified regions where similar neural activity was predicted by semantic similarity. They found that spoken discourse on similar topics elicited similar activation patterns in a widely distributed and bilateral brain network. This bilateral network was more extensive but overlapped with regions where similar activation was associated with similar topics during comprehension. Semantic similarity effects were bilateral, even while univariate activation contrasts of these data were left-lateralized. This result suggests that the right hemisphere homologues of the language network encode semantic properties even when they do not show significant activation over baseline. The authors concluded that right hemisphere homologues have a supportive role in processing the meaning of discourse during comprehension and production.

Another study evaluated inter- and intra-hemispheric connectivity in processing unambiguous versus semantically ambiguous words (homophonic homographs, such as bark on a tree and bark of a dog, and heterophonic homographs, such as bass the fish vs. bass the instrument) in neurotypical adults. For heterophonic homographs, they observed increased connectivity within the left hemisphere, indicating top-down re-activation of orthographic representations by phonological representations to process alternative meanings. For homophonic homographs, they showed bidirectional flow of information from left to right and from right to left, indicating a greater role of the right hemisphere in understanding these words [30].

### 2.3. The Role of the Cerebellum

Several recent reviews have discussed neuroanatomical and functional imaging evidence for a strongly lateralized involvement of the right cerebellum in a variety of nonmotor (as well as motor) language functions through functional and structural connections between the right cerebellum and language cortex [31,32,33]. The right cerebellum is at least involved in monitoring and coordinating functions of the cortical language network. Many functional imaging studies of language show activation of the right or bilateral cerebellum as well as the right hemisphere homologues of the language network, although these areas are often not mentioned in the text [34]. A recent coordinate-based meta-analysis of the language processing of 403 experiments found that language primarily engaged the bilateral fronto-temporal cortices, with the highest activation in the left pIFG but also the left fusiform gyrus (FuG), bilateral auditory, and left postcentral regions. Importantly, they also found strong bilateral subcortical and cerebellar contributions. The right cerebellum was activated during a variety of speech production and visual and phonological language tasks [35].

### 2.4. The Language Networks and Supporting Areas: Summary

This brief review of evidence from language processing in neurotypical individuals supports the view that there exists a reliable left-hemisphere cortical language network that includes the superior, middle, and inferior temporal cortex, Fu, pIFG, DLPFC, and IPC and their connections. Additionally, there are left hemisphere areas that seem to be frequently engaged in language that may have a supportive role, including the superior frontal gyrus (which includes the supplementary motor area (SMA) and the pre-supplementary motor area (pre-SMA)), the cingulate gyrus, and their connections, especially with the IFG. Additionally, both right hemisphere homologues of the language network and the right cerebellum play critical supporting roles in neurotypical individuals. We propose that these areas and their connections might be potentiated to help recover language after stroke. Furthermore, connections between undamaged language network nodes and these supporting regions can be strengthened to support recovery. In the next section, we review imaging studies of language recovery in post-stroke recovery that provide some support for this type of remapping underlying aphasia recovery.

## 3. Imaging Recovery via the Pre-Existing Right-Hemisphere Language Network

The dominant underlying mechanisms driving aphasia recovery are thought to shift over time after stroke. Acute functional recovery is attributable to restoration of local blood flow in perilesional (i.e., surrounding) tissue [36,37,38]. Over time, the mechanisms of recovery shift. Subacute recovery is supported by increased activation of the right-hemisphere language network [39] and driven by lesion extent and location within the left hemisphere [7]. That is, while spared ipsilateral perilesional tissue plays a key role [40,41] where left-hemisphere language network tissue is damaged, homologous contralateral regions are engaged to a greater degree. If the entire left hemisphere is damaged, the right MTG, SMG, and AG become most active in language [42].

More selective lesions are associated with more restricted right hemisphere engagement. For example, a meta-analysis contrasting those with and without lesions in the left IFG demonstrated that in those for whom the left IFG was preserved, activation of the right frontal areas was limited to the anterior pars triangularis and MTG [9]. However, in those for whom the left IFG was damaged, right-sided activation extended from the pars triangularis to the dorsal pars opercularis, pars orbitalis, and pre- and post-central gyrus. Irrespective of IFG lesion, activation of the right ventral pars opercularis and left MFG was noted. Sebastian et al. longitudinally examined four participants with naming deficits following stroke in the posterior cerebral artery (PCA, which does not supply the traditional language network, so these areas were structurally intact) using task-based and resting-state functional MRI [43]. During language tasks, participants generally demonstrated robust activation of the bilateral language network, even when measured acutely. Language recovery from the acute to chronic phase was associated with greater balance of left- and right-dominant activation within the language network and its homologues.

Language recovery in aphasia is supported further by domain-general processes that arise from a bilateral network [44,45,46]. Because language tasks are presumably more difficult for people with disordered language than those without, there may be greater activation of regions supporting attention and cognitive control during language tasks in people with aphasia than in those without. This can lead to ambiguity about how to best interpret bilateral frontal activation in people with aphasia. However, taken together, there is relative consensus that recovery of language involves the right STG and likely the right SMA, middle frontal gyrus, precentral gyrus, AG, MTG, temporal pole, pSTS, precuneus, insula, and anterior cingulate cortex [41], reflecting both domain-specific and domain-general regions.

Multiple studies have observed changes in bilateral and right hemisphere homologous network connectivity associated with functional improvement following treatment of aphasia. For example, in one trial, naming impairment was associated with poor coherence of low frequency BOLD fluctuations within and across the ipsilesional left and contralesional right language cortex at the acute stage after PCA stroke, and functional connectivity improved over time only in participants who showed good naming recovery [43]. Another trial contrasted pre- and post-treatment connectivity and found that pre-treatment fluctuations in BOLD signal and synchrony of fluctuations across regions (amplitude of low-frequency fluctuations) measured in the right MTG were associated with greater treatment response [47]. In the same sample, post-treatment fluctuations in the left MTG and STG and right IFG were associated with greater treatment response. Treatment was associated with restored connectivity between the left MTG and STG and between the right and left IFG. Connectivity of the right pars triangularis [48] and bidirectionally between the right pars triangularis and left fusiform gyrus [49] have been associated more specifically with recovery of concrete words.

However, sustained, greater than normal interhemispheric connectivity is not a positive sign for all individuals when considering all paired regions and functions. The complex landscape of changing function and changing activation is only beginning to be disentangled [50]. However, the granular knowledge of these systems will be crucial to individualizing treatment and predicting outcomes in future individuals. Predictably, it is the extent to which connectivity is preserved at baseline that significantly predicts treatment outcomes (in fMRI [51], EEG [52,53,54], and in functional near-infrared spectroscopy [55]). While acute interhemispheric connectivity in stroke survivors with language deficits is below that of normal age-matched adults, the magnitude of change can reflect an over-correction or “hyper-normalization” and can be negatively correlated with functional improvement. For example, greater magnitude of increased functional connectivity between the right and left dorsal frontoparietal and dorsal prefrontal areas has been associated with *poorer* response to treatment of spelling [56]. However, the authors note that connectivity after treatment was not associated with poorer accuracy (just a smaller change in accuracy), arguing against a maladaptation interpretation of their findings. In an electroencephalographic dynamic causal modeling study, *reduced* coupling between the right IFG and pSTG was associated with the best recovery [53]. Consistently, normal-like levels of connectivity within a left-dominant language network result in optimal levels of function and the greatest improvement [14,57,58]. This association is also found when examining global measures of network fidelity [59,60] and dynamics [61,62].

These observations add nuance to our understanding of the right-hemisphere language network’s role in functional recovery. Studies converge in showing that the best recovery is generally seen when the normal, left-hemisphere language network is adequately spared such that enhanced dependence on the right homologous network is not needed. However, when the normal left-hemisphere language network is sufficiently damaged such that normal or compensatory intrahemispheric connectivity cannot be restored, at least part of the right homologous network is often recruited to support language recovery.

## 4. Treatments Aimed to Engage Supportive Areas or Connections to Promote Recovery

### 4.1. Treatments Thought to Engage Right Homologous Network

Various intervention strategies for aphasia are thought to stimulate the right-hemisphere language network, such as those that incorporate music, musical techniques, and drawing. Often, multimodal approaches are introduced in combination to provide communication intervention and support for people with aphasia. Studies have also explored methods involving experimental manipulations to attention and intention, as well as neurostimulation of the right cerebellum, with the aim of improving outcomes. While not all of these approaches have been employed sufficiently broadly and diversely to generate the highest quality evidence of their efficacy (e.g., clinical trials of individual strategies and subsequent meta-analyses), taken together, they provide an important line of evidence for the utility of incorporating right-hemisphere dominant tasks in language treatment.

### 4.2. Music-Based Treatments

Music-based approaches incorporate such elements as intoned speech, melodic contour, metrical timing, rhythmic tapping, and unison production and are broadly aimed at facilitating speech output by improving one’s speech fluency [63,64]. Treatment protocols for aphasia involving music and musical techniques include Melodic Intonation Therapy (MIT [65]), Speech Music Therapy for Aphasia [66], SIPARI^®^ [67], and other music-based methods that incorporate singing, melody, and rhythm [64,68]. MIT—which has the largest research evidence base of the music-based intervention approaches for aphasia— integrates melody via varied intonation and rhythm via left-hand tapping during verbal expression [69]. During MIT, the participant is guided to produce a slower rate of articulation with continuous voicing, which is thought to reduce dependence on the left hemisphere and engage the right hemisphere. The participant is also guided to tap their left hand, which is thought to provide pacing and continuous cueing for syllable production and to engage the sensorimotor network in the right hemisphere [69,70]. Treatment progresses along hierarchies of token complexity and clinician support, initially beginning with two-syllable words/phrases and greater clinician support and advancing to longer phrases with less or no clinician support [70].

In terms of behavioral outcomes in people with aphasia, reviews of MIT report positive effects on participants’ word and sentence repetition ability, story retelling, and phrase length, with smaller effects seen in measures of functional, everyday communication and variable effects seen in measures of comprehension [71,72,73] One group [73] conducted a review of MIT clinical trials that included imaging and found that the right hemisphere brain regions activated by MIT included areas of the frontal motor cortex, including the pIFG, auditory cortex (including the STG and MTG), and the parietal cortex (including the angular gyrus and gyrus). Another study [63] found evidence of changes in activation in various right hemisphere regions, including the pSTG, pIFG, inferior pre-central gyrus, postcentral gyrus, pre-SMA, and SMG, following participation in MIT. In reviews of other music-based interventions, improvements in speech outcomes, such as word and sentence repetition, and language outcomes, such as improved conversational informativeness, are noted [74,75]. Interestingly, individuals with co-occurring aphasia and motor speech deficits seem to benefit more from music-based interventions compared to participants with aphasia without co-occurring motor speech deficit. This may suggest a motor-speech-based mechanism of improvement [64].

### 4.3. Drawing

Drawing is another modality used in aphasia interventions that is thought to engage the right hemisphere. While drawing often serves as an alternative, compensatory means of communication for people with aphasia (i.e., in lieu of verbal speech in moments of anomia), it is also used as a treatment element in multimodal, restorative treatment approaches designed to facilitate improvements in verbal speech. Drawing is thought to facilitate a different level of semantic processing and a different approach to accessing one’s semantic system by increasing the person’s attention to an object’s structural and perceptual characteristics—or in other words, its visual features [76,77]. This differs from other modalities such as writing, which relies on a lexical route to phonological output and engages the left hemisphere [78]. Drawing has been found to increase accurate naming in significantly more instances than writing [76,78]. Relatedly, fMRI studies have shown that in a group of people with aphasia, drawing produces stronger activation in the right hemisphere compared to writing, indicating that drawing differentially engages the brain compared to a linguistically-based task like writing [76,79]. When drawing an object, its semantic features are activated, which is thought to potentially eliminate semantic competitors that do not share semantic features with the target and to subsequently facilitate target retrieval and production [78]. Additionally, it has been proposed that the fixed nature of drawn symbols may facilitate success in retrieving or activating an object’s name by serving as a non-transient representation of the underlying concept [80].

Systematic reviews assessing the effectiveness of drawing in improving language outcomes are limited in number, primarily because drawing is typically integrated as one of several components within multimodal treatments for aphasia. Consequently, these reviews cannot parse out the unique contributions of drawing on improvements seen in language outcomes following such multi-modal treatment approaches. Alongside gesturing and writing, drawing is one of the modalities included in Multi-Modality Aphasia Therapy [81], Promoting Aphasics’ Communicative Effectiveness [82], and the ongoing clinical trial for treating subacute-chronic post-stroke aphasia via telemedicine, PICTURE IT (NCT05845047). Reports examining the effectiveness of multimodal approaches that include drawing combined with semantic feature cueing and other communicative modalities (e.g., gesturing) generally report improvements in naming [83]. Case reports and treatment studies that have isolated drawing as the sole element of treatment, such as Back to the Drawing Board [84] and Functional Drawing Training [85], primarily aim to increase people with aphasia’s use of drawing as a means of communication (e.g., in the case of severe expressive aphasia) or to improve their drawing ability/quality, and thus, the extent to which such approaches result in improvements in the more standardized, impairment-based language outcomes is not clear.

### 4.4. Attention and Intention Treatments

Manipulations to attention and intention have also emerged as promising strategies to engage the right hemisphere during language tasks. Manipulating spatial attention during naming/treatment activities, by directing attention to the left visual space, is hypothesized to transfer language function to the right hemisphere [86]. Several studies have demonstrated that placing stimuli in the left hemispace, which may be engaging spatial attention mechanisms in the intact right hemisphere, can improve people with aphasia’s language performance [86,87,88]. Intention treatments aim to shift the lateralization of language production to right frontal structures by incorporating complex left-hand movements that engage the pre-SMA area [89]. A number of studies have reported that performing complex, multi-stage movements with the left-hand during naming tasks results in improved naming accuracy and can lead to higher concentrations of activity in the right frontal lobe following the treatment [90,91,92]. In one study [93], the investigators compared naming outcomes in a cohort of 34 people with moderate to profound aphasia following both attention and intention treatment conditions. They found that all participants showed significant improvements in naming following both treatment conditions; however, the rate of improvement was greater in the intention treatment condition for those with moderate and severe aphasia. These findings underscore the potential that attention and intention manipulations can enhance recovery outcomes.

### 4.5. Non-Invasive Brain Stimulation

Non-invasive brain stimulation (NIBS) techniques most commonly refer to the application of repetitive transcranial magnetic stimulation (rTMS) or transcranial direct current stimulation (tDCS), though transcranial alternating current stimulation (tACS) has also been explored [94]. In contrast to the behavioral approaches to aphasia rehabilitation reviewed thus far, NIBS may be applied concurrently with (theoretically) any behavioral approach in the hope of enhancing the therapeutic benefit due to the physiological effects of neurostimulation on synaptic plasticity (that is, generating or inhibiting action potentials). While TMS and tDCS are applied using differing devices and, subsequently, have differing safety profiles, the underlying physiological mechanism of proposed augmentation is comparable. One way in which strategies for applying NIBS differ beyond stimulation site is in whether they apply inhibitory stimulation to the homologous regions in the right hemisphere or excitatory stimulation to the ipsilateral, ideally preserved regions. There are multiple systematic reviews of the literature on the efficacy of NIBS in the treatment of aphasia [95,96,97,98]. These meta-analyses generally conclude that there is a small but measurable augmentative effect of NIBS, though it may vary due to individual factors (e.g., genetics, age) and lesion characteristics [99,100].

An example application of neurostimulation that provides unique insight into the present discussion of the right-hemisphere language network is the application of tDCS to the right cerebellum. Two studies investigating the efficacy of neuromodulation to the right cerebellum have demonstrated that pairing right cerebellar transcranial direct current stimulation (tDCS) with behavioral treatment may be a promising avenue through which to augment behavioral treatment outcomes. In one study, a participant who had sustained bilateral strokes and was experiencing anarthria participated in a course of therapy in which right cerebellar tDCS (initially a sham condition followed by an active condition) was coupled with behavioral spelling therapy [101]. Results included significant improvements in the participant’s spelling accuracy (to dictation) for both trained and untrained words following both conditions; however, improvements were greater in the active tDCS condition compared to the sham condition. Notably, improvements in spelling accuracy for untrained words and generalization to written picture naming were exclusively observed following the active tDCS condition [101]. Furthermore, imaging results indicated increased cerebro-cerebellar resting state functional connectivity following treatment, suggesting potential modifications to the underlying networks supporting spelling as a result of right cerebellar tDCS. In another study, a group of 21 participants with chronic post-stroke aphasia participated in a randomized, double-blind, sham-controlled, within-subject crossover design experiment in which the right cerebellar tDCS (again, either sham or active) was coupled with a computerized program of word picture matching [102]. Similar to the findings from the case study, improvements in the outcome for untrained targets were only seen following the active condition. These findings suggest that tDCS over the right cerebellum (with concomitant behavioral treatment) enhances language recovery compared to sham stimulation. Additionally, it appears to increase connectivity between the right cerebellum and the right and left language networks as well as within the right and left language networks.

## 5. Conclusions

Here we have reviewed studies that have shown that a network of right hemisphere areas homologous to the language network and the right cerebellum have a supportive role in language in neurotypical individuals. We have also reviewed evidence that some people with aphasia remap language to these supportive areas or show increased functional connections between these areas and left-hemisphere language network as they recover language. Finally, we discussed behavioral interventions designed to engage the right hemisphere to promote language recovery using music, drawing, gesture, attention, or pragmatics. Other studies have shown the benefit of stimulating the right cerebellum to increase connections between the cerebellum and language network areas in both hemispheres to augment aphasia recovery. Together, these studies indicate that one successful approach to language improvement is to augment remapping of language to the right hemisphere, or right cerebellar–cortical connections.

## Abbreviations

AG	Angular gyrus
DLPFC	Dorso-lateral prefrontal cortex
FuG	Fusiform gyrus
IFG	Inferior frontal gyrus
IPC	Inferior parietal cortex
MTG	Middle temporal gyrus
Pre-SMA	Pre-supplementary motor area
SMA	Supplementary motor area
SMG	Supramarginal gyrus
STG	Superior temporal cortex
STS	Superior temporal sulcus
